# A retrospective study on improving the accuracy of radiotherapy for patients with breast cancer with lymph node metastasis using Styrofoam

**DOI:** 10.2478/raon-2024-0001

**Published:** 2024-01-06

**Authors:** Jie Li, Lin Yang, Xiaowei Yao, Linlin Xu, Lina Zhao, Fei Bai

**Affiliations:** Department of Radiation Oncology, Xijing Hospital, Fourth Military Medical University. Xi’an, China

**Keywords:** breast cancer, Styrofoam, setup error, planning target volume

## Abstract

**Background:**

To retrospectively analyze the accuracy of radiotherapy using cone beam computed tomography (CBCT), Styrofoam fixation, and breast bracket fixation in the chest wall target area and supraclavicular lymphatic drainage area (supraclavicular target area) of patients with breast cancer.and compare the setting efficiency and comfort satisfaction.

**Patients and methods:**

A total of 65 patients with postoperative lymphatic metastasis of breast cancer, including 36 cases of Styrofoam fixation and 29 cases of breast bracket fixation, were recruited from March 2021 to August 2022 and retrospectively analyzed. All the patients underwent CBCT scans weekly, and the setup errors of the chest wall and supraclavicular target volume were compared and recorded. The planning target volume (PTV) margins of the two groups were calculated using the correlation M_PTV_ = 2.5Σ + 0.7σ. The setup time and comfort satisfaction scores of the two groups were recorded and analyzed. The correlations among errors in each direction were analyzed using the Pearson correlation analysis.

**Results:**

There was a significant difference in the left-right direction (X) axis of the chest wall target area between the Styrofoam and breast bracket groups (1.59 ± 1.47 mm *vs.* 2.05 ± 1.64 mm, P = 0.012). There were statistical differences in the ventrodorsal direction (Z) and bed angle of the supraclavicular target area, the data were (1.36 ± 1.27 mm *vs.* 1.75 ± 1.55 mm, P = 0.046; 0.47 ± 0.47° *vs.* 0.66 ± 0.59°, P = 0.006, respectively). In the X, Y, and Z directions, the respective PTV margins of the two groups in the chest wall target area were 5.01 mm, 5.99 mm, and 5.47 mm in the Styrofoam group, while those in the breast bracket group were 6.10 mm, 6.34 mm, and 6.10 mm, respectively. Moreover, the PTV margins of the supraclavicular target in the three directions were 3.69 mm, 3.86 mm, and 4.28 mm in the Styrofoam group, while those in the breast bracket group were 3.99 mm, 3.72 mm, and 5.45 mm, respectively. The setup time of the two groups was 3.4 ± 1.1 min and 5.5 ± 3.1 min (P = 0.007). The subjective comfort satisfaction scores of the two groups were 27.50 ± 1.24 and 25.44 ± 1.23 (P < 0.001).

**Conclusions:**

The application of Styrofoam fixation in radiotherapy of breast cancer in the supraclavicular lymph node area has several advantages as compared to breast bracket fixation, including higher positioning accuracy, smaller external expansion boundary, improved work efficiency, and patients’ comfort, which might provide a reference for clinical work.

## Introduction

Since the 21st century, the incidence and mortality rates of female breast cancer have shown an overall increasing trend. In 2020, the rate of female breast cancer reached the top of the global cancer incidence spectrum and global female cancer death spectrum.^1^ In China, a total of 420,000 new breast cancer cases have been reported in 2020.^2^ Breast cancer is treated by a combination of different treatment strategies, including surgery, radiation therapy, chemotherapy, and endocrine therapy. The 10-year local recurrence rate in axillary node-positive patients is 46%, which can be decreased to 13% with postoperative radiotherapy.^3^ The intensity-modulated radiation therapy (IMRT) and cone-beam computed tomography (CBCT) have shown significant improvements with the advancements in radiotherapy technology. IMRT can achieve arbitrary dose distribution for breast cancer and improve dose uniformity of the irradiated area.^4,5^ Using imaging, the CBCT technology can guide radiation to accurately irradiate within the target area of the breast tissues.^6^

In breast cancer radiotherapy, the lungs and heart are the main organs at risk (OARs). Reducing the dose to these organs can reduce the radiation-induced long-term cardiovascular and lung damage.^7,8^ Accurate setup is one of the methods to reduce the exposure dose to OARs under the premise of advanced radiotherapy technology and Radiation Therapy Oncology Group (RTOG) guidelines. The accuracy of setup can be improved by selecting the professional level of therapists along with the optimization of position selection and position fixation mode during radiotherapy. The role of the therapists becomes increasingly important in this dynamic environment.^9^ Zhou *et al.*^10^ showed that fixation of the chest wall with a vacuum bag required CTV-PTV margins (PTV margin) of 12, 12.37, and 14.25 mm in X, Y, and Z directions, respectively, while 10.71 mm, 10.91 mm, and 13.87 mm margins were required for the supraclavicular target area. Dong *et al.*^11^ found that when the fixation device with arm support was used, the PTV margins of 8.14 mm, 10.89 mm, and 6.29 mm were required in X, Y, and Z directions, respectively, while in the case of using cervicothoracic thermoplastic membrane combined with arm support, the PTV margins of 8.01 mm, 5.44 mm, and 5.45 mm were required in X, Y, and Z directions, respectively. Svestad *et al.*^12^ positioned patients with breast cancer using WingSTEP™ and found that the patients needed margins of 5 mm, 10 mm, and 8 mm in the three directions. Mulliez *et al.*^13^ compared two positioning systems, including Positrest-2 system (Civco Medical Solutions, Orange City, Ia, USA) in a supine position and AIO prone breast system (AIO Solution, Orfit Industries, Wijnegem, Belgium) in a prone position. The results showed that 9.4 mm, 9.4 mm, and 10.4 mm margins were required in the three directions for patients in a supine position, while 22.4 mm, 13.7 mm, and 10.5 mm margins were required in a prone position. It could be seen that the standard PTV margin of 5 mm in the chest wall target area in a fixed position was insufficient both in the supine and prone position under the existing vacuum bag, arm support, and cervicothoracic thermoplastic membrane fixation mode. similar results were also observed for the supraclavicular lymphatic drainage area (supraclavicular target area). The repeatability of the neck and arm directly affects the accuracy of supraclavicular target irradiation as well as effects of radiotherapy. Currently, there are limited studies on supraclavicular target setup errors in breast cancer radiotherapy with a smaller sample size as compared to the chest wall, and the setup error of the conventional fixed method is larger. Therefore, it is necessary to develop and explore good repeatability of the fixation device. Styrofoam fixation (registered name: Body Positioning Mats, Guangzhou Fury, China) has the advantages of individualization and high comfort, and it has been confirmed in other tumor radiotherapy position fixation.^14,15^ Therefore, the current study focused on investigating the repeatability of the supraclavicular target irradiation setup of Styrofoam in patients with breast cancer.

This study retrospectively analyzed the setup error of each target volume in breast cancer radiotherapy fixed by Styrofoam glue using CBCT and compared it with the fixation device with arm support (Abbreviation: breast bracket). The setup repeatability and PTV margin of the two fixation devices were also compared. The current study also provided a reference basis for the fixation device with good repeatability for breast cancer radiotherapy target areas, including the supraclavicular lymphatic drainage area.

## Patients and methods

### Case selection

This was a single-institution retrospective study, which was approved by the institutional ethics committee (KY0202002-F-1). The study included the patients, receiving IMRT radiotherapy after breast cancer surgery between March 2021 and August 2022. The inclusion criteria included (1) patients with breast cancer confirmed by pathology, (2) patients with postoperative radiotherapy target, including supraclavicular lymphatic drainage area, (3) patients with good abduction and upper limb lifting function of the affected side, and (4) patients with KPS (Karnofsky) score greater than 80. The exclusion criteria were as follows: (1) the patients receiving chest wall radiotherapy only, (2) the patients with difficulty in upper arm support and unable to meet fixation, and (3) the patients who were unwilling or unable to complete the whole study process.

### Patients’ CT positioning and treatment plan formulation

**FIGURE 1.**
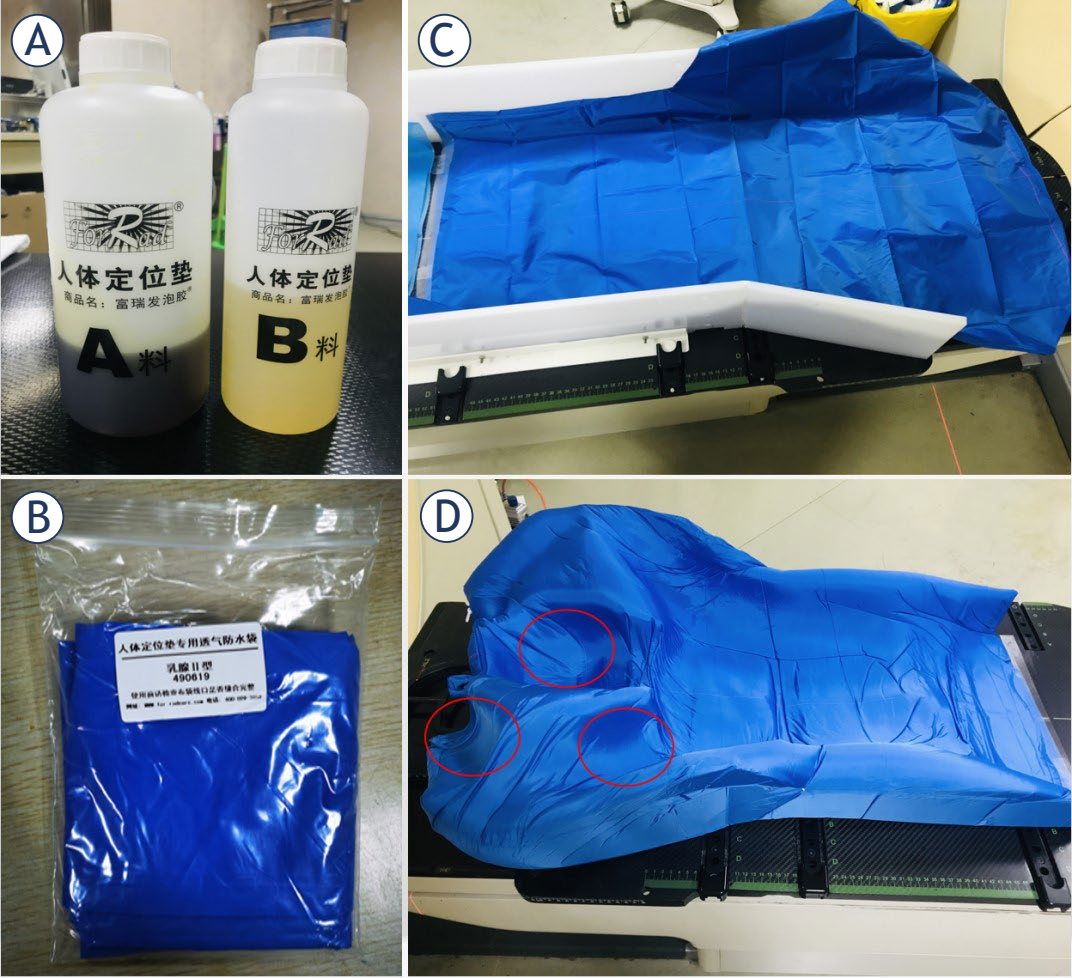
Making process of Styrofoam. **(A)** Glue. **(B)** Bags. **(C)** Unformed Styrofoam. **(D)** Making of molded Styrofoam (Individualized head fixation as well as arm fixation as shown in the red circle)

According to the fixation mode (due to the introduction of Styrofoam postural fixation of breast cancer in routine practice in 2022, the previous fixation was terminated), the selected patients were divided into two groups, including the breast bracket and Styrofoam glue groups. The production process of Styrofoam is shown in Figure 1. In both groups, the mandible of the participants was raised as much as possible, their heads were inclined to the healthy side by 15 degrees, and their arms were raised naturally. The 16-slice large-aperture spiral Computer Tomography (CT) was used to simulate localization in the two groups. The scanning range was 5 cm from submandibular to subdiaphragmatic with a slice thickness and interval of 5 mm. The scanned images were sent to the doctor’s workstation system, and the radiotherapist delineated the target area in the CT positioning image combined with other image data based on the RTOG standard, limited the surrounding organs at risk, and formulated a target dose. The CTV was enlarged to 5 mm to form a PTV. The sketched images were sent to the radiotherapy treatment planning system (TPS), and the radiotherapy plan was prepared by the radiotherapy physicist. The radiotherapy plan was transferred to the accelerator after verification.

### CBCT image acquisition and matching

**FIGURE 2.**
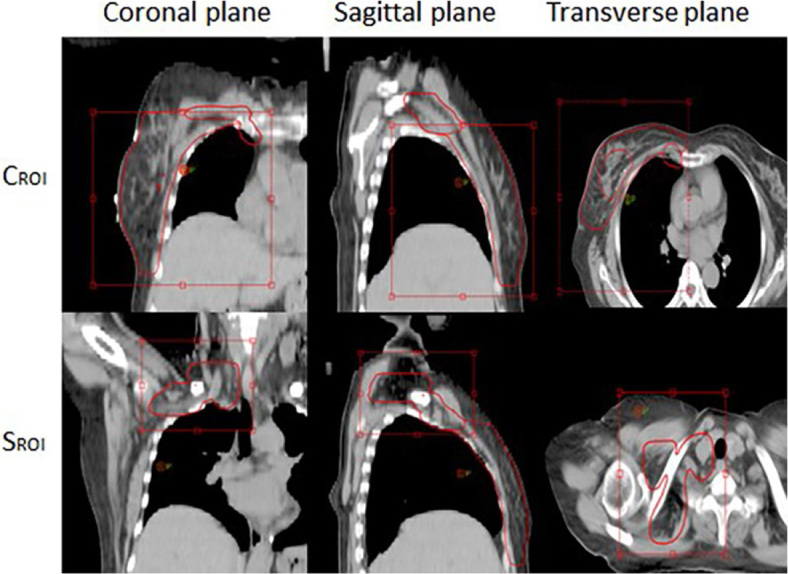
ROI Matching. CROI = chest wall target; SROI = supraclavicular target

The CBCT images were obtained using the on-board image guidance system (OBI) of the Clinac IX linear accelerator purchased from Varian Medical Systems, California, US. The regions of interest were placed in the chest wall and s supraclavicular target area, respectively, as shown in Figure 2. The CBCT image was compared with the positioning CT planning image. Then, the setup errors of the X-axis (left and right), Y-axis (head and foot), Z-axis (front and rear), and the foot of table (RTN) were calculated. Moreover, the error data was recorded after manual adjustment according to the target area position.

### CTV-PTV margin

According to International Commission on Radiation Units and Measurements (ICRU) reports 50 and 62, there should be a certain distance outside the CTV to reduce the setup error and effects of patient and tissue motion on the target volume. In our institution, Clinicians will follow unified standard for delineation and will delineate an ITV to address the effects of organ motion. For the setup error, a uniform 5 mm external margin is adopted. In this study, we mainly study the CTV (Including ITV)-PTV margin caused by setup error. The marginal calculation formula of Van Herk *et al.*^16^ was used in this study to ensure that at least 95% of the prescribed dose was given to 90% of the patients with CTVs. The marginal calculation formula is given in Eq. (A).

(A) MPTV=2.5Σ+0.7δ

where the group systematic error (Σ) and random error (δ) were the standard deviations of individual systematic and random errors, respectively.

### Comparison of setting efficiency and comfort satisfaction

The setup time for each fraction of the two fixation techniques was recorded and counted. The time required for the setup was defined as the time when the patient sat on the treatment couch until the therapist walked out of the treatment room after the setup. Each patient was investigated weekly. Subjective comfort A questionnaire survey was conducted for the first treatment to understand patients’ subjective comfort satisfaction with the device. The satisfaction survey comprised eight items, each with a 5-point Likert scale. The dimensions of evaluation included head, neck, and back comfort, mask fit, tightness, temperature, color, anxiety about the fixture, general discomfort, and recommendation of the fixture.

### Statistical analysis

The data were analyzed using SPSS25.0 statistical software. The data counts were expressed as frequencies (n) and percentages (%). The measurement data were expressed as mean ± SD. All the setup errors were taken as absolute values, and the data results were expressed as mean ± standard deviation (x ± s). Between the two groups, the differences in data counts were compared using the χ^2^ test, while all the other comparisons were performed using t-tests. The correlation of errors in each direction was analyzed using the Pearson correlation analysis. A P-value of < 0.05 was considered statistically significant.

## Results

Among 78 patients with breast cancer who received IMRT, 65 patients met the inclusion criteria. Among these 65 patients, 36 patients received Styrofoam fixation, and 29 patients received bracket fixation. A total of 281 CBCT verifications, including 147 Styrofoam and 134 breast bracket verifications, were performed. The clinical data of the two groups were analyzed, and the results are listed in Table 1. No significant differences between the indices were observed (P > 0.05).

**TABLE 1.** Comparison of clinical data between the two groups

**Group**	**Styrofoam n = 36**	**Bracket n = 29**	**P-value**
**Age (x ± s, years)**	48.34 ± 9.58	48.90 ± 10.92	0.837
**Affected Side (n, %)**			0.565
**Left**	20 (55.6)	11 (37.9)	
**Right**	16 (44.4)	18 (62.1)	
**Type of operation (n, %)**			0.053
**Bcs**	7 (16.7)	9 (31)	
**Rm**	29 (83.3)	20 (69)	
**Stage (n, %)**			0.146
**II**	7 (19.4)	4 (13.8)	
**III**	26 (72.2)	20 (68.9)	
**IV**	3 (8.4)	5 (17.3)	

BCS = Breast Conserving Surgery; RM = Radical Mastectomy

### Setup error

The setup error of the Styrofoam glue in the chest wall target area in the left-right direction was less than the breast bracket (1.59 ± 1.47 mm *vs.* 2.05 ± 1.64 mm, t = 2.516, P = 0.012), while that in the supraclavicular target area in the abdominal-dorsal direction was less than the breast bracket (1.36 ± 1.27 mm *vs.* 1.75 ± 1.55 mm), t = 2.003, P = 0.046. Moreover, the couch angle error of Styrofoam rubber was less than that of the breast bracket (0.47 ± 0.47° *vs.* 0.66 ± 0.59°), t =2.760, P = 0.006. The detailed results are provided in Table 2.

**TABLE 2.** Comparison of setup errors of the two fixation methods in the chest wall target area and supraclavicular target area (mm,`x ± s)

**Group**	**Styrofoam**	**Bracket**	**t**	**P-value**
**CROI (X)**	1.59± 1.47	2.05 ± 1.64	2.516	0.012
**(Y)**	1.99 ± 1.46	2.10 ± 1.59	0.611	0.541
**(Z)**	1.78 ± 1.47	2.00 ± 1.58	1.235	0.218
**SROI (X)**	1.23 ± 0.88	1.32 ± 1.16	0.620	0.536
**(Y)**	1.23 ± 1.21	1.16 ± 1.17	−0.445	0.657
**(Z)**	1.36 ± 1.27	1.75 ± 1.55	2.003	0.046
**CRTN (°)**	0.48 ± 0.46	0.53 ± 0.43	1.033	0.302
**SRTN (°)**	0.47 ± 0.47	0.66 ± 0.59	2.760	0.006

CROI = Chest Wall Target; SROI = supraclavicular target

### PTV margin comparison

In the breast bracket group, the PTV margins of the chest wall in the X, Y, and Z directions were 6.10 mm, 6.34 mm, and 6.10 mm, respectively, while those of the supraclavicular target area were 3.99 mm, 3.72 mm, and 5.45 mm, respectively. In the Styrofoam group, the PTV margins of the chest wall in the X, Y, and Z directions were 5.01 mm, 5.99 mm, and 5.47 mm, respectively, while those of the supraclavicular target area were 3.69 mm, 3.86 mm, and 4.28 mm, respectively (Table 3). In the chest wall target area, the margin of the Styrofoam group was 17.87% smaller than that of the bracket group in the X direction. In the supraclavicular target area, the margin of the Styrofoam group was 21.47% less than that of the bracket group in the Z direction.

**TABLE 3.** Target volume expansion boundary in the three-dimensional direction in 65 patients in the breast bracket and Styrofoam groups (mm)

		**Styrofoam**			**Bracket**		

		**X**	**Y**	**Z**	**X**	**Y**	**Z**
**Σ Systematic error**	CROI	1.59	1.99	1.78	2.05	2.10	2.00
	SROI	1.23	1.23	1.36	1.32	1.16	1.75
**σ Random error**	CROI	1.47	1.46	1.46	1.39	1.57	1.57
	SROI	0.88	1.12	1.26	0.98	1.17	1.54
**M_PTV_**	CROI	5.01	5.99	5.47	6.10	6.34	6.10
	SROI	3.69	3.86	4.28	3.99	3.72	5.45

Note: ∑ was the standard deviation of the systematic error of each patient, and the systematic error of each patient in each direction was the average value of the errors in each direction among all fractions; σ was the mean square value of the random error of each patient, and the random error of each patient in each direction was the standard deviation of the error in each direction among all fractions; and MPTV was the size of the outspread boundary from the clinical target volume to the planned target volume.

### The comparison of the displacement frequency of the chest wall target area

**FIGURE 3.**
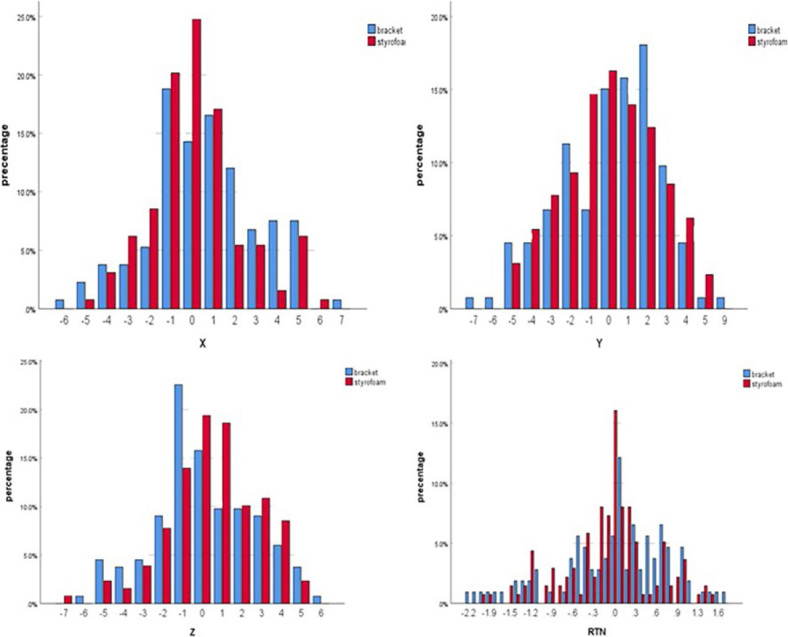
Bar chart of setup error of two fixation methods in chest wall target area.

The displacement distributions for < 3 mm, 3~5 mm, and > 5mm in the Styrofoam group in the X direction were 75.4%, 23.1%, and 1.5%, respectively, and those in the Y direction were 66.8%, 32.1%, and 1.1%, respectively. In the Z direction, the displacement distributions for the respective frequencies were 69.2%, 29.2%, and 1.6%. In the breast bracket group, the displacement distributions for <3 mm, 3~5 mm, and > 5mm in the X direction were 66.9%, 31.6%, and 1.5%, respectively, while those in the Y direction were 66.9%, 30.8%, and 2.3%, respectively. Moreover, in the Z direction, the displacement distributions for the respective were, 66.9%, 31.6%, and 1.5%, respectively. The probability of < 3 mm of Styrofoam in the left and right direction was significantly greater than that of the bracket group, as shown in Figure 3.

### A comparison of the displacement frequency in the supraclavicular target area

**FIGURE 4.**
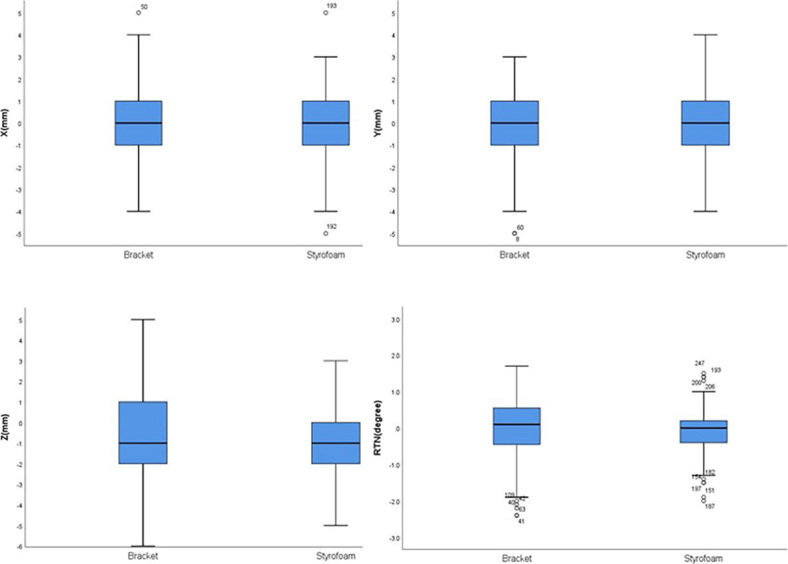
Box plot of setup error in supraclavicular region of two fixation methods.

The displacement distributions for < 3 mm, 3~5 mm, and > 5mm in the Styrofoam group in the X-direction were 89.7%, 9.1%, and 1.2%, respectively, and those in the Y-direction were 84.5%, 15.5%, and 0%, respectively. In the Z-direction, the displacement distributions of respective were 82.8%, 17.2%, and 0%. The displacement distributions for < 3 mm, 3~5 mm, and > 5mm in the breast bracket group in the X-direction were 87.3%, 10.8%, and 1.9%, respectively. In the Y-direction, the displacement distributions were 87.7%, 12.3%, and 0%, while in the Z-direction, they were 76.5%, 20.6%, and 2.9%, respectively, as shown in Figure 4.

### Comfort satisfaction score of a fixation device and comparison of setup efficiency

The subjective comfort satisfaction scores of patients in the Styrofoam and bracket groups were 27.50 ± 1.24 and 25.44 ± 1.23 points, respectively, showing a statistically significant difference (P < 0.001). The setup times of the Styrofoam and breast bracket groups were 3.4 ± 1.1 min and 5.5 ± 3.1 min, respectively (P = 0.007).

### Correlation analysis of setup error between two groups of different directions

**FIGURE 5.**
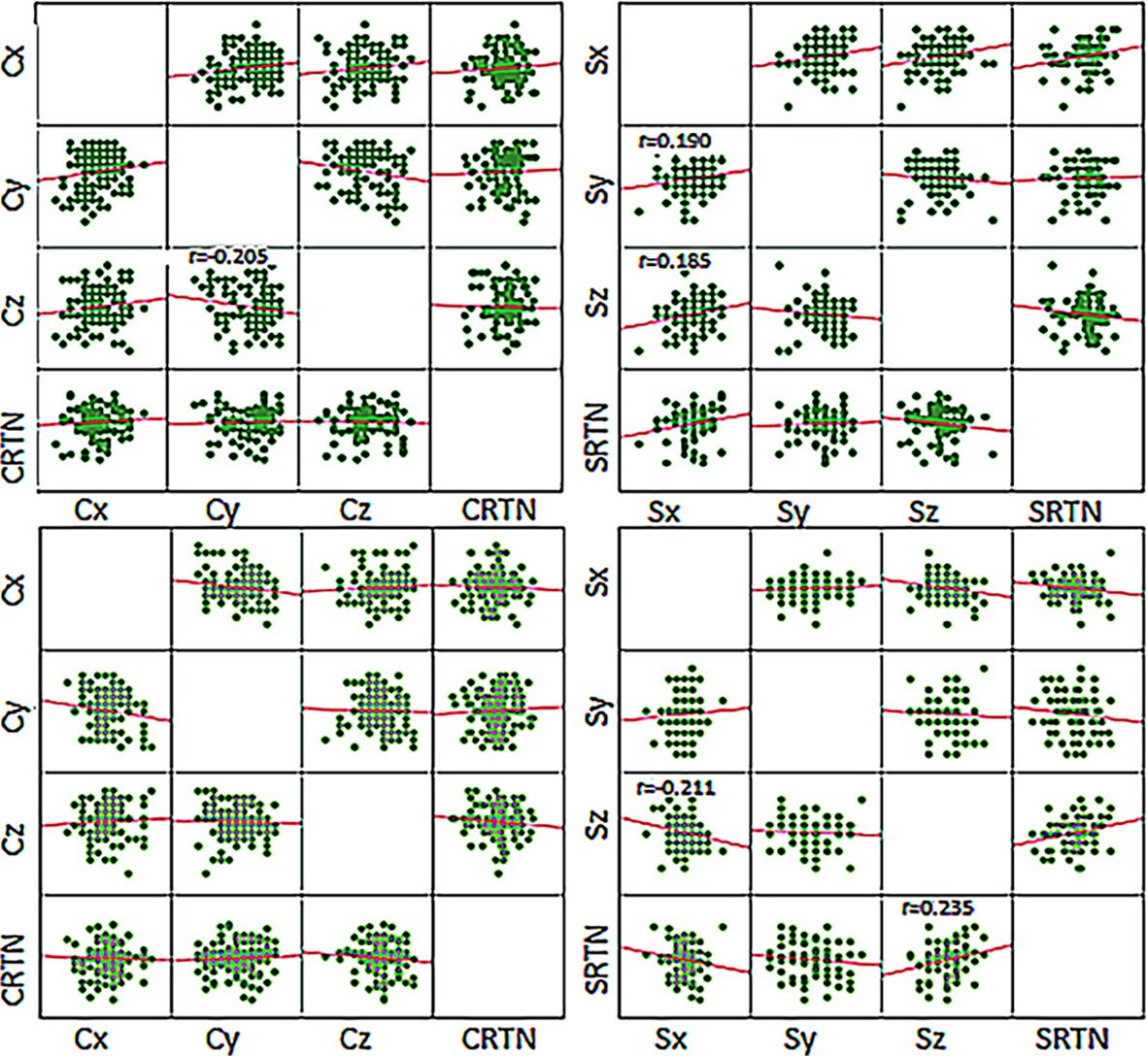
Scatter plot of setup error between two groups in different directions. The dark color in the upper figure shows the bracket group, and the light color in the lower figure shows the Styrofoam group. The r-values with P-values < 0.05 are indicated in the figure. C = chest wall; S = supraclavicular

In the bracket group, the Pearson correlation analysis showed a moderate correlation between the Y-axis and Z-axis direction in the chest wall setup error (r = −0.205), while the supraclavicular target area X-axis setup error showed a weak correlation with the Y and Z-axis directions (r = 0.190 and 0.185). The Z-axis setup error in the Styrofoam group supraclavicular target area was moderately correlated with the X-axis direction and RTN (r = −0.211 and 0.235), as shown in Figure 5.

## Discussion

The current study compared the Styrofoam fixation and breast bracket fixation in patients with breast cancer, who underwent postoperative radiotherapy. The results showed that Styrofoam fixation could significantly reduce inter-fractional displacement in the X direction of the chest wall and displacement in the Z and RTN directions of the supraclavicular region. The chest PTV margin of the foam group was 17.87% (5.01 mm *vs.* 6.10 mm) less than that in the bracket group in the left and right directions. In the supraclavicular region, the Styrofoam group was 21.47% (4.28 mm *vs.* 5.45 mm) less exposed in the anteroposterior direction as compared to the bracket group. The Styrofoam fixation group showed a higher comfort satisfaction score and work efficiency.

The accuracy of radiotherapy directly affects the success or failure of radiotherapy.^17,18^ The setup error was relatively large due to the special physiological structure of breast cancer. Errors in breast cancer radiotherapy are related to factors, such as fixation devices, the patient’s position, experience of the radiotherapy therapist, and the patient’s body mass index.^19^ When the fixture is more comfortable, it reduces the setup error more effectively. Currently, the commonly used molds for breast cancer positioning in various radiotherapy centers include vacuum bags, thermoplastic body films, breast brackets, and Styrofoam. A vacuum bag poses a risk of air leakage and compression deformation during treatment. The thermoplastic phantom could significantly reduce inter-fraction error in IMRT for breast cancer as compared to a vacuum bag. However, it might increase the irradiated skin dose at the irradiated site, thereby exacerbating radiation skin reactions.^20,21^ Therefore, care should be taken while using thermoplastic masks for fixation. In the breast bracket (a conventional mechanical fixing device), the fixation of the neck and shoulder was uncertain, and it was easier to form a forced body position. Moreover, the repeatability of the clavicle area could not be guaranteed, and the degree of individualization of mold was not as high as that of Styrofoam.

Our results showed that the Styrofoam group had a smaller setup error than the bracket group in the X direction of the chest wall. Zhou C *et al.*^22^ compared the vacuum bag and Styrofoam fixation in 40 patients after breast-conserving radical mastectomy for breast cancer and revealed that the setup errors in the X, Y, and Z directions of the Styrofoam group were 1.63 ± 1.29 mm, 1.46 ± 1.51 mm, and 1.30 ± 1.35 mm, respectively, which were less than those of the vacuum bag group (1.83 ± 1.61 mm, 2.26 ± 2.03 mm, and 1.91 ± 1.67mm, respectively). In their study, the Styrofoam and vacuum pad groups showed similar results to those of the Styrofoam and bracket groups in the current study in terms of errors in the X and Y directions errors. Fang Jiannan *et al.*^23^ conducted a study on 24 patients treated with breast-conserving radiotherapy for breast cancer and showed that the Styrofoam rubber group exhibited a smaller setup error as compared to that of the breast bracket group in the Y direction (1.76 ± 1.78 mm *vs.* 3.28 ± 2.79 mm), X direction (2.36 ± 2.89 mm *vs.* 2.56 ± 2.05 mm), and Z direction (1.47 ± 1.49 mm *vs.* 1.73 ± 1.81 mm) with higher work efficiency. However, in the current study, the Styrofoam group showed smaller setup errors in the X direction while having similar setup errors in other directions. The differences between the two studies might be due to differences in sample size, treatment procedures in the respective centers, and experience levels of the therapists (the therapists in the current study had an experience of more than 10 years). In conclusion, the above studies demonstrated that the Styrofoam fixation could significantly improve the setup accuracy, repeatability, and setup efficiency of the fixation and might be a promising individualized fixation device.

This study found that Styrofoam fixation in the supraclavicular region had a significantly smaller setup error in the Z direction as compared to that in the bracket group. Zhang Y *et al.*^24^ used cervicothoracic membrane in combination with breast bracket for fixation in 32 patients with breast cancer after the operation, and the respective setup errors on the supraclavicular target area were 1.98 ± 2.44 mm, 1.98 ± 2.48 mm, and 1.71 ± 1.79 mm. Except for the bracket group, which had similar setup errors in the Z orientation, the setup errors of other orientations were significantly greater than those of any of the fixation devices in this study. Shen K *et al.*^25^ used vacuum bag fixation in 24 patients with radiotherapy after mastectomy and revealed that the standard deviations of the setup error on the supraclavicular target area were 1.6 mm, 1.4 mm, and 1.8 mm, which were similar to those of the bracket group in the current study. Based on the previous studies, it could be observed that Styrofoam fixation in the supraclavicular target area might have less error, which might be because the Styrofoam is an individualized fixation device, and the patient’s comfort satisfaction scores were higher (27.50 ± 1.24 *vs.* 25.44 ± 1.23 points). In this study, individualized fixation was also performed on the patient’s arm, and the arm showed better plasticity on Styrofoam, as shown in Figure 1 D. The patient could quickly and accurately repeat positioning on his fixation device. The setup efficiency can also reflect (3.4 ± 1.1 *vs.* 5.5 ± 3.1) min.

The PTV margins of the IMRT target volume in most studies were 5 mm. The current study showed that the calculated PTV margins of the target volume fixed by Styrofoam in the X, Y, and Z directions of the chest wall were 5.01 mm, 5.99 mm, and 5.47 mm, respectively, while those of the supraclavicular target area were 3.69 mm, 3.86 mm, and 4.28 mm, respectively. Moreover, in the breast bracket group, the chest wall boundaries of the calculated PTV margins were 6.10 mm, 6.34 mm, and 6.10 mm in the three directions, while the supraclavicular margins were 3.99 mm, 3.72 mm, and 5.45 mm, respectively. It could be seen that for both fixation devices, a PTV margin of 5 mm was not sufficient on the chest wall, while it was sufficient for the supraclavicular target area. Yao W *et al.*^26^ fixed 25 patients with breast cancer with Styrofoam and showed that the corresponding margins were 6.75 mm, 8.46 mm, and 8.73 mm. In comparison, the chest wall margins in the current study were smaller. Shen K *et al.*^25^ showed that the chest wall systematic errors in the X, Y, and Z directions were 1.67 mm, 2.37 mm, and 1.31 mm with random errors of 1.70 mm, 1.83 mm, and 1.68 mm, respectively, while the supraclavicular target area systematic errors in the three directions were 1.02 mm, 0.90 mm, and 1.19 mm with the random errors of 1.22 mm, 1.20 mm, and 1.44 mm, respectively. The calculated PTV margins for the chest target area were 5.36 mm, 7.20 mm, and 4.46 mm, while those for the supraclavicular target area were 3.41 mm, 3.08 mm, and 3.98 mm, respectively. Zhang Y *et al.*^24^ The calculated PTV margins were 6.2 mm, 6.7 mm, and 5.7 mm in the chest wall target area, while those in the supraclavicular target area were 6.6 mm, 6.7mm, and 5.5 mm. The PTV margin required for the set-up error in this study is slightly smaller than in other studies, which might be due to differences in the fixation devices and standards in different studies. Similarly, the differences in sample size and other factors could not be excluded. As a radiotherapy therapist, in this study, the CTV-PTV margins caused by set-up error was discussed, and in the ICRU report62, clinicians will delineate the ITV based on factors such as organ movement.

The current study showed that the setup errors of the two groups of fixation devices in the Y direction of the chest target area contained more positive values; this indicated that the position of the two groups of patients moved to the foot side. In addition, the couch angle in both groups had extreme values, which might be due to a slightly greater number of patients enrolled after radical mastectomy on the right side. During the late-course treatment, the patient’s arm could not be naturally lifted to the original positioning position due to irradiation and surgery, and the body was affected by traction to shift to the affected side, resulting in coronal rotation. Therefore, the radiotherapist must educate patients with breast cancer to do functional exercises of the affected upper limb after radiotherapy. The data in the supraclavicular target area showed more negative values in the Z direction in both groups, suggesting that both groups collapsed in the neck region. A similar phenomenon was also reported by Svestad JG *et al.*^12^ However, in addition to the patient in the CT positioning of the body is too tight and the body relaxed during treatment reasons, cannot rule out the therapist in the setup process is not rigorous and caused by human error, radiation therapists should avoid this problem.

The current study analyzed the setup errors in all directions using the Pearson correlations analysis, which has been rarely studied in previous studies. The correlation analysis was used to analyze whether an increase in error in one direction would change the error in the other direction. The results showed that the setup error of bracket fixation in the Y direction in the chest wall region was negatively correlated with that in the Z direction, while the setup error in the X direction in the supraclavicular target area was weakly correlated with that in the Y and Z directions; this was also consistent with clinical practice. When the X direction error became larger, the side deformed the neck in the headrest, resulting in changing the neck error in the Y and Z directions. In the Styrofoam group, the setup error in the Z direction of the supraclavicular target area was negatively correlated with that in the X direction, while it was positively correlated with the RTN. This might be due to the relatively stronger head fixation of Styrofoam (Figure 1 D), and the error in the Z direction, which caused the coronal rotation of the neck. This also showed that the bracket fixation was not as good as the Styrofoam fixation for the patient’s head.

The current study has several limitations. First, only two groups of fixation methods were discussed, and there were many factors, which affected the setup error. Secondly, van Herk’s boundary calculation was performed only for the errors of three horizontal displacements. At larger target volumes, even small rotational errors can lead to dose uncertainty.^27^ In clinical practice, it is difficult to correct rotational errors and local setup errors using CBCT image guidance. However, a combination of a six-dimensional treatment couch^28^ and an optical surface detection system (OSMS)^29^ can be used to solve these problems in a qualified unit. Finally, the effects of breathing on positioning were not explored in this study and are needed to be studied further in the future. The use of artificial intelligence (AI) in radiotherapy is increasing; therefore, it is expected to apply AI techniques for error correction. Mathematical models or computerized deep learning might help in reducing the setup errors of breast cancer in the future. It is also possible to quantify the physical indicators, including weight and body mass index, which will be the subject of future studies. Future studies with larger sample sizes, multifactorial setup errors, and better fixation methods are needed.

In summary, the current study retrospectively analyzed the use of Styrofoam fixation in radiotherapy for patients with lymph node metastasis after breast cancer surgery. The study suggested that the use of Styrofoam could further improve the setup accuracy and setup efficiency of the chest wall and supraclavicular target area, improve patients’ comfort and satisfaction, and decrease the PTV margin distance. This study might provide a reference for the clinical use of Styrofoam glue to fix the postoperative radiotherapy of patients with breast cancer.
